# Social Media Exposure, Psychological Distress, Emotion Regulation, and Depression During the COVID-19 Outbreak in Community Samples in China

**DOI:** 10.3389/fpsyt.2021.644899

**Published:** 2021-05-12

**Authors:** Yu-ting Zhang, Rui-ting Li, Xiao-jun Sun, Ming Peng, Xu Li

**Affiliations:** ^1^Key Laboratory of Adolescent Cyberpsychology and Behavior Central China Normal University (CCNU), Ministry of Education, Wuhan, China; ^2^School of Psychology, Central China Normal University, Wuhan, China; ^3^Department of Psychiatry, Renmin Hospital of Wuhan University, Wuhan, China

**Keywords:** COVID-19, social media exposure, depression, psychological distress, emotion regulation

## Abstract

The outbreak of coronavirus disease 2019 (COVID-19) has been a global emergency, affecting millions of individuals both physically and psychologically. The present research investigated the associations between social media exposure and depression during the COVID-19 outbreak by examining the mediating role of psychological distress and the moderating role of emotion regulation among members of the general public in China. Participants (*N* = 485) completed a set of questionnaires online, including demographic information, self-rated physical health, and social media exposure to topics related to COVID-19. The Impact of Event Scale-Revised (IES-R), the Beck Depression Inventory-II (BDI-II), and the Emotion Regulation Questionnaire (ERQ) were utilized to measure psychological distress about COVID-19, depression, and emotion regulation strategies, respectively. Results found that older age and greater levels of social media exposure were associated with more psychological distress about the virus (*r* = 0.14, *p* = 0.003; *r* = 0.22, *p* < 0.001). Results of the moderated mediation model suggest that psychological distress mediated the relationship between social media exposure and depression (β = 0.10; *Boot 95% CI* = 0.07, 0.15). Furthermore, expressive suppression moderated the relationship between psychological distress and depression (β = 0.10, *p* = 0.017). The findings are discussed in terms of the need for mental health assistance for individuals at high risk of depression, including the elderly and individuals who reported greater psychological distress and those who showed preference usage of suppression, during the COVID-19 crisis.

## Introduction

The outbreak of coronavirus disease 2019 (COVID-19), a severe acute respiratory syndrome (SARS), was reported on December 31, 2019, in Wuhan, China. Within several weeks, the disease had rapidly spread throughout the world, and on March 9, 2020, the World Health Organization (WHO) declared that COVID-19 had turned into a worldwide pandemic (1). By May 11, 2020, more than 4 million individuals worldwide had been diagnosed with COVID-19 (2), and the number of cases is still on the rise.

Previous research has demonstrated noticeable psychological problems in individuals diagnosed with COVID-19 (3, 4) as well as the general public (5–7). In a study conducted in hospitalized patients diagnosed with COVID-19, it was estimated that approximately one third of patients with COVID-19 experience symptoms of anxiety and depression, with symptom severity being associated with lower social support (4). In another study, more than half of health care workers reported symptoms of depression, with greater severity among frontline health care workers who worked directly with patients (8). Moreover, due to the highly contagious nature of the disease, strict lockdown was imposed all over China. The COVID-19 crisis has also had a significant impact on the mental health of members of the general public, people who have not become ill because of the virus may nevertheless experience psychological distress related to the illness. In a nationwide survey of 52,730 non-patients in China at the end of January 2020, about 35% of individuals reported experiencing moderate to severe psychological stress related to COVID-19 (9). More specifically, the prevalence rates of depression were 20.1% in Huang and Zhao (10) and 53.5% in Liu et al. (11), estimated with the Center for Epidemiology Scale for Depression [CES-D; (12)] and the Patient Health Questionnaire-9 [PHQ-9; (13)], respectively. Approximately 4.6% of participants suffered from posttraumatic stress symptoms 1 month after the COVID-19 outbreak (14).

Beyond establishing prevalence, it is important to identify factors associated with higher and lower risk of depression among the general population during the COVID-19 pandemic. Massive social media use was found to be associated with poor sleep quality, elevated depressive symptoms, and behavior issues in adolescents, such as cyberbullying (15–17). Previous research demonstrated that greater exposure to trauma-related media information was associated with an increased risk of developing mental health problems over time. In the study of Holman et al. (18), they compared the impact of media-based indirect exposure and direct exposure on acute stress response after 2013 Boston Marathon bombing, and it was found that bombing-related media exposure was more strongly related to acute stress than direct exposure to the bombings (18), and these associations may accumulate over time, generating a vicious cycle of media use and distress (19).

According to the emotional contagion theory (20), emotional state could be transferred from one person to another through automatic mimicry, such as facial expression and postures. For example, happiness can be spread from person to person through social interactions (21). Moreover, emotional contagion could also occur online, in the absence of typical in-person interaction clues (22, 23), especially for negative emotions. Negative posts were followed by more negative responses than positive posts on Twitter, which then increased the amount of negative posts the following week and thus provided greater opportunity for the emotional contagion (24). Media effect theory has been developed to explain how media use brings a change to people's cognition, emotion, and behavior (25).

A great deal of information outrushed on the Internet after the outbreak of COVID-19. Internet posts concerning COVID-19 showed a sharp increase after human-to-human transmission was confirmed on January 20, 2020, and the number of posts was associated with the number of diagnosed patients (26), indicating great concern about the spread of COVID-19. Though health information could help relieve the stress (27), misinformation was also disseminated, and it may cause fear and stress among the public (28). According to the emotional contagion theory and media effect theory, those who did not get infected of the virus may also suffer from emotional distress and depression after browsing social media posts related to COVID-19. Consistently, several studies have demonstrated that massive social media exposure to information related to COVID-19 was positively associated with more severe mental health problems, such as anxiety and depression (29, 30). Nevertheless, only a few studies have examined the underlying mechanism that might mediate or moderate this association. Liu and Liu (31) found that exposure to social media was related to higher levels of anxiety, and the association was mediated by vicarious traumatization. Given the close relationship between social media exposure and perceived distress (18, 19, 31), the present study assumed that psychological distress may play a mediation role between social media exposure and depression.

People use multiple emotion regulation strategies to regulate their emotional response to crisis. Cognitive reappraisal involves the cognitive reevaluation of emotion-inducing situations. The use of cognitive reappraisal can reduce negative affect and its physiological correlates, thus it is considered to be an adaptive emotion regulation strategy (32). In addition, the use of cognitive reappraisal was associated with higher levels of positive affect and greater satisfaction with life (33–35) and better psychological consequences such as decreased anxiety and depression [e.g., (36)]. Expressive suppression is a response-focused form of emotion regulation when a person tries to inhibit his or her emotion expressive behavior after the emotional response has already been generated (32). Expressive suppression is considered a maladaptive emotional regulation strategy, which has been shown to increase negative emotional feelings and result in poor social consequences (37). Generally, expressive suppression was associated with higher and cognitive reappraisal with lower posttraumatic symptoms in response to crisis (38, 39), while another study reported a non-significant correlation between cognitive reappraisal and severity of posttraumatic symptoms in a clinical sample of trauma-exposed women (40).

There are only a few studies that examine the interaction between stress and emotion regulation on psychological well-being, and mixed results have been reported. Roos et al. (41) found that suppression, rather than reappraisal, moderated the relationship between stressful life events and physiological responses to acute stressors, while another study suggested a moderating role of cognitive reappraisal between stress and depression (42). Nevertheless, in a recent study using daily diary method, it was found that both cognitive reappraisal and expressive suppression moderated the associations between stress and suicidal thoughts, and the associations were weaker among individuals who reported habitual use of either strategy (43).

While previous studies have investigated psychological distress and depression severity related to COVID-19 separately, to the best of our knowledge, no study has examined the extent to which emotion regulation strategies may predict or moderate relations between psychological distress and depression during the COVID-19 outbreak. Given the high prevalence rate of depression on the public under COVID-19 (11), assessing the moderating role of emotion regulation between psychological distress and depression may uncover the mechanism of generating and developing mental illness during the pandemic and provide evidence for the effectiveness of applying certain emotion regulation strategies on reducing mental health burden among the general population.

The present study was conducted in mid-February 2020, at which time the number of COVID-19 cases in China had reached 66,576 (44), and the number was still rising. The sample was made up of members of the general population who were not patients with COVID-19. The goals of the study were to estimate the prevalence of depression and to explore the relationships among social media exposure, psychological distress about COVID-19, emotion regulation strategies, and symptoms of depression. Social isolation is helpful in preventing virus spread but also could be a public health concern for the elderly (45) and was a risk factor for depression and anxiety (46). Therefore, it was hypothesized that (1) the elderly would report more severe mental health problems and (2) social media exposure may exacerbate psychological distress and depression during the COVID-19 outbreak. Considering that adaptive and non-adaptive emotion regulation strategies could be utilized in responding to stress elicited by COVID-19 and were closely related to severity of depressive symptoms, moderation analyses were conducted to examine whether the use of emotion regulation moderated the predictive relationship between psychological distress and depressive symptom. As there is still much controversy regarding the moderating effect of specific emotion regulation strategies on the relations between psychological distress and depression (38, 41, 42), no specific hypothesis was made regarding the moderating role of suppression and reappraisal. The moderating role of suppression and reappraisal would be examined, respectively.

## Methods

### Participants

Potential participants among Chinese citizens were invited to complete questionnaires *via* the Internet, using links sent *via* Social Networking Services (SNSs; such as WeChat) from February 16 to February 19, 2020, using a snowball sampling technique. Of the 576 participants who filled out the questionnaires, 87 were excluded from the final data analysis because the completion time was <180 s or the same answer was given to more than 80% of the items. Four participants were diagnosed patients or frontline medical workers and were also excluded from analysis. There were 485 participants in the final sample (193 males, 39.8%; 292 females, 60.2%). Participants' ages ranged from 12 to 75, with most (76.1%) aged between 18 and 50. Nearly half of the participants (45.8%) were currently enrolled students. About half lived in urban areas (212; 43.7%) and about half in rural areas (273; 56.3%). About half were married, divorced, or widowed (226; 46.6%) and about half were single (259; 53.4%). Among the participants, 55 (11.3%) were from Hubei province. This study was approved by the local ethics committee. All participants provided informed consent to having their anonymous data used for research. In addition, informed consent was obtained from teachers of middle school students before data collection.

### Measures

#### Demographic Information

Demographic variables included age, gender (male, female), marital status (single, married, divorced, widowed), education level (middle school, high school, college or higher), and region (urban, rural). In addition, participants were asked to provide a self-rating of physical health on a 5-point Likert scale from 1 (“very bad”) to 5 (“very good”).

#### Coronavirus Disease 2019-Related Information

Social media exposure was measured by one item, which was consistent with a previous study (29). Participants rated how much they focused on information related to COVID-19 on social media (e.g., Weibo, WeChat) each day using a 5-point Likert scale from 1 (“almost never”) to 5 (“almost always”).

#### Psychological Distress

The Impact of Event Scale-Revised [IES-R; (47); Chinese version by (48)] is a frequently used self-report scale to measure psychological distress following a traumatic event (49). The IES-R contains 22 items, and participants are asked to rate each item on a 5-point Likert scale ranging from 0 (“not at all”) to 4 (“extremely”), resulting in a total possible score ranging from 0 to 88. The items were adapted to refer in particular to distress elicited by COVID-19. For example, the original item “Any reminder brought back feelings about it” was changed to “Any reminder brought back feelings about COVID-19.” The Cronbach α coefficient in the present study was 0.92.

#### Depression Severity

The Beck Depression Inventory-II [BDI-II; (50)] was used to measure depressive symptoms. The BDI-II contains 21 items. On each item, participants are asked to choose one of four statements that best describes their feelings, with scores ranging from 0 to 3 for each item. For example, one item provides the following four options: “I do not feel sad” (0), “I feel sad” (1), “I am sad all the time and I can't snap out of it” (2), and “I am so sad and unhappy that I can't stand it” (3). The total possible score ranges from 0 to 63, and participants can be categorized as being at one of four levels of depression severity according to their total score: no or minimal depression (0–13), mild depression (14–19), moderate depression (20–28), and severe depression (≥29). The Chinese version of BDI-II was reliable on assessing depressive symptom (51). The Cronbach α coefficient in the present study was 0.92.

#### Emotion Regulation

Participants' use of various emotion regulation strategies was measured using the Emotion Regulation Questionnaire [ERQ; (32)]. The ERQ includes 10 items, and participants are asked to rate each item on a 7-point Likert scale ranging from 1 (“strongly disagree”) to 7 (“strongly agree”). The ERQ has two subscales: cognitive reappraisal (six items) and expressive suppression (four items). A higher subscale score indicates more frequent use of that emotion regulation strategy. The Chinese version of ERQ was proven to be good in reliability and validity (52). In the present study, the Cronbach α coefficients were 0.88 and 0.76 for the cognitive reappraisal subscale and expressive suppression subscale, respectively.

### Data Analysis

**Figure 1:**
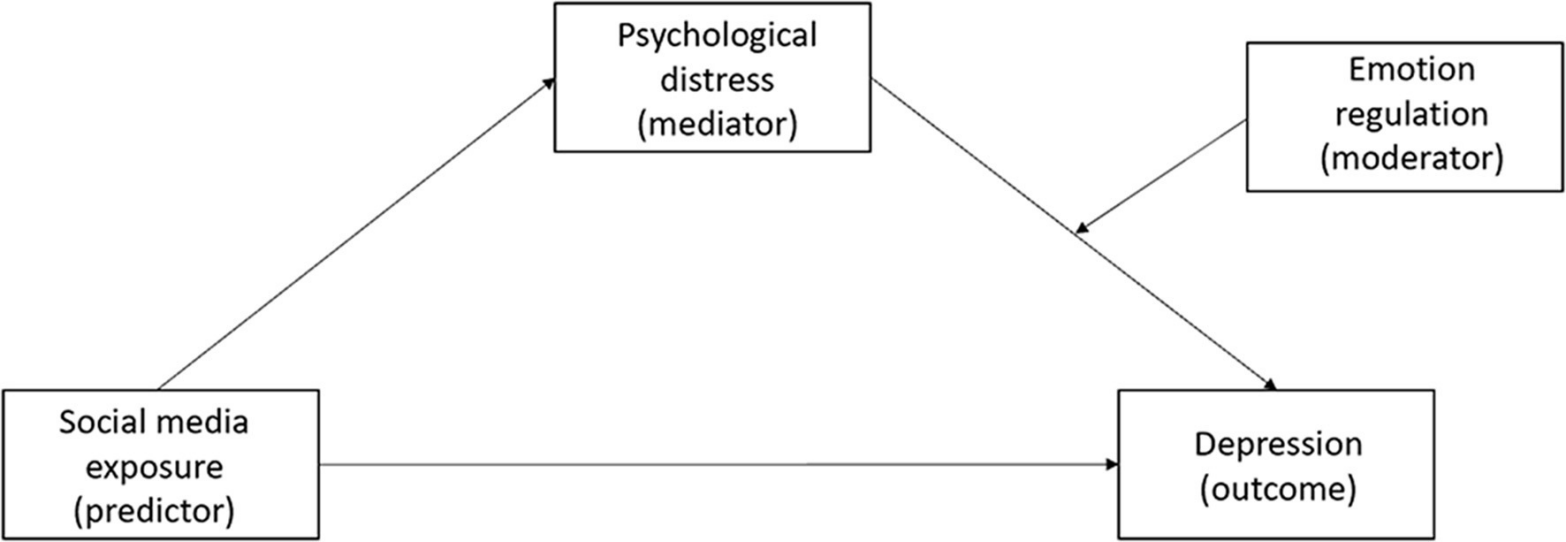
The hypothesis moderated mediation model of social media exposure, psychological distress, emotion regulation, and depression.

Data analyses were conducted using SPSS 25.0, and the *p*-value threshold for statistical significance was set at 0.05 (two-tailed). First, to establish the validity of the data, common method bias was assessed using Harman's single-factor test. Principal component analysis extracted 10 factors whose eigenvalues were larger than 1, and the first factor explained 23.36% of the total variance. Result did not reveal severe common method bias in the present study. Then, descriptive analyses were conducted, including correlations among all variables. Independent-samples *t*-tests and one-way analyses of variance (ANOVAs) were conducted to determine if scores for depression and for psychological distress about COVID-19 varied depending on demographic variables, physical health, and social media exposure. The prevalence of depression was also estimated. Secondly, a moderated mediation model was conducted using Model 14 of PROCESS macro (53) to further explore the relationship of social media exposure, psychological distress, emotion regulation strategies, and depression (Figure 1). The first step of direct regression of independent variable to dependent variable was not necessary for mediation analysis (54); thus, the full model was conducted straightforward. Additionally, conditional direct and indirect effects were calculated with non-parametric bootstrapping method with 5,000 resamples. Finally, simple slope analysis was conducted to explore the patterns of significant moderation effect.

## Results

### Descriptive Information

The ANOVA results showed that individuals at an older age and those with a higher education level experienced more severe psychological distress than individuals at a younger age or with a lower level of education (see Table 1 for descriptive and test statistics). Additionally, there was a significant positive correlation between age and psychological distress, *r* = 0.14, *p* = 0.003. Self-rated health was associated with depression and psychological distress; individuals with worse physical health status suffered more severe depression and psychological distress about the virus.

**Table 1:** Comparison of sample characteristics on psychological distress and depression.

**Characteristic**		***n* (%)**	**IES-R**	**BDI-II**
			***M* ±*SD***	***M* ±*SD***
Full sample		485 (100)	21.63 ± 13.55	6.24 ± 8.00
Gender	Male	193 (39.8)	21.46 ± 13.69	6.19 ± 8.99
	Female	292 (60.2)	21.74 ± 13.48	6.28 ± 7.30
	*t*		0.22	0.12
Region	Urban	212 (43.7)	22.65 ± 13.97	5.66 ± 6.91
	Rural	273 (56.3)	20.84 ± 13.18	6.69 ± 8.75
	*t*		1.46	1.45
Locality	Hubei province	55 (11.3)	23.62 ± 13.71	7.20 ± 5.85
	Others	430 (88.7)	21.38 ± 13.52	6.12 ± 8.24
	*t*		1.156	0.943
Age (years)	①>20	133 (27.4)	18.46 ± 13.53	7.09 ± 8.90
	②21−30	142 (29.3)	22.46 ± 13.11	6.34 ± 7.42
	③31−40	93 (19.2)	22.60 ± 12.92	6.14 ± 8.64
	④41−50	96 (19.8)	23.24 ± 14.52	5.41 ± 7.39
	⑤50>	21 (4.3)	24.48 ± 12.39	4.48 ± 5.06
	*F*		2.68^*^	0.90
	Bonferroni		① < ④^a^	
Education	①Middle school	147 (30.3)	17.42 ± 12.71	6.44 ± 8.41
	②High school	95 (19.6)	22.15 ± 13.45	6.66 ± 8.12
	③College or higher	243 (50.1)	23.98 ± 13.53	5.95 ± 7.73
	*F*		11.30^**^	0.33
	Bonferroni		① < ②, ① < ③	
Marital Status	①Married^b^	226 (46.6)	22.92 ± 13.60	5.48 ± 7.31
	②Unmarried	259 (53.4)	20.51 ± 13.43	6.90 ± 8.52
	*T*		1.96	1.96
Self-rated health	①Bad or average	60 (12.4)	22.87 ± 14.38	9.88 ± 10.33
	②Good	144 (29.7)	25.24 ± 13.65	6.85 ± 7.20
	③Very good	281 (57.9)	19.52 ± 12.92	5.15 ± 7.59
	*F*		9.05^**^	9.58^**^
	Bonferroni		② > ③	① > ②, ① > ③

*IES-R, The Impact of Event Scale-Revised; BDI-II, Beck Depression Inventory-II*.

a *p = 0.08*.

b *Including married, divorced, and widowed*.

* *p <0.05*,

** *p <0.01*.

Descriptive statistics and correlations among social media exposure, psychological distress, emotion regulation, and depression are presented in Table 2. Social media exposure was positively related to psychological distress and depression, *r* = 0.22, *p* < 0.001; *r* = 0.09, *p* = 0.042. Psychological distress was positively correlated with depression, *r* = 0.45, *p* < 0.001. Significant correlations were also found between the use of the expressive suppression emotion regulation strategy and psychological distress, *r* = 0.22, *p* < 0.001, and depression severity, *r* = 0.16, *p* < 0.001. The correlations between cognitive reappraisal and depression or psychological distress were not significant, *p*s > 0.05.

**Table 2:** Descriptive statistics and correlations among psychological distress, emotion regulation, and depression.

	**1**	**2**	**3**	**4**	**5**	***M***	***SD***
1.Social media exposure	–	0.22^**^	0.09^*^	0.02	−0.01	3.93	0.90
2. IES-R		–	0.45^**^	0.07	0.22^**^	21.63	13.55
3. BDI-II			–	−0.03	0.16^**^	6.24	8.00
4. ERQ: cognitive reappraisal				–	0.54^**^	27.87	7.36
5. ERQ: expressive suppression					–	15.20	4.77

*N = 485. IES-R, The Impact of Event Scale-Revised; BDI-II, Beck Depression Inventory-II; ERQ, Emotion Regulation Questionnaire*.

* *p < 0.05*,

** *p < 0.01*.

### Prevalence of Depression

The prevalence of depression was estimated based on the BDI-II categorical system (50). In the current sample, 413 participants (85.1%) were classified as showing no to minimal depression (BDI-II scores from 0 to 13); 39 participants (8.0%) showed mild depression (BDI-II scores 14–19); 24 participants (5.0%) showed moderate depression (BDI-II scores 20–28), and nine participants (1.9%) showed severe depression (BDI-II scores 29 and above). Thus, 15.9% of the sample showed at least mild depression according to the BDI-II system of classifying respondents according to the severity of depression.

### The Moderated Mediation Model

**Figure 2:**
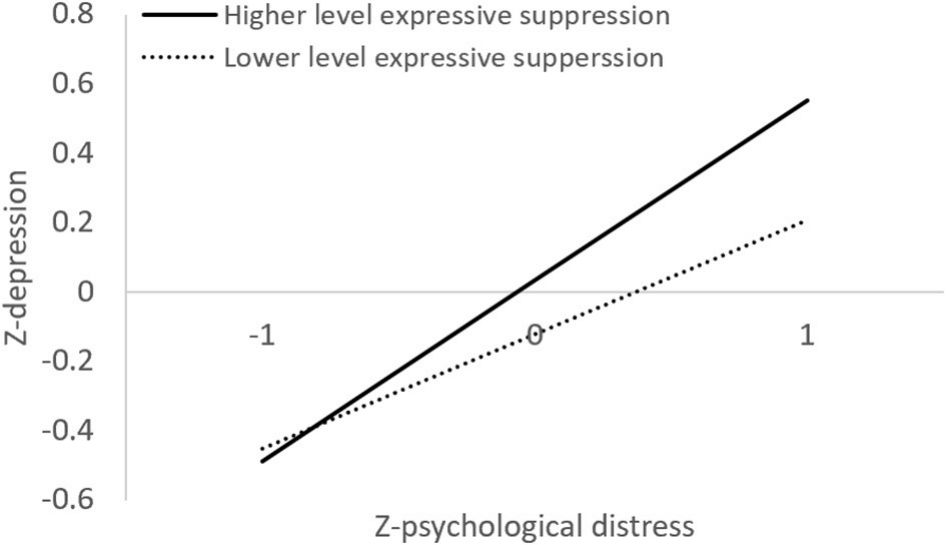
Illustration of the moderating effect of expressive suppression on the relationship between psychological distress and depression.

To examine the relationship between social media exposure, psychological distress, emotion regulation, and depression, a moderated mediation model was conducted. Results showed that social media exposure positively predicted psychological distress (β = 0.24, *p* < 0.001), and psychological distress positively predicted depression severity (β = 0.043, *p* < 0.001; Table 3). The conditional indirect effect was significant (β = 0.10; *Boot 95% CI* = 0.07, 0.15), while the conditional direct effect was non-significant (β = −0.04; *Boot 95% CI* = −0.12, 0.05). Thus, these results indicated that psychological distress fully mediated the relationship between social media exposure and depression. In addition, the interaction of psychological distress and expressive suppression in predicting depressive symptoms was significant (β = 0.10, *p* = 0.017). Simple slope analysis showed that among individuals who reported higher frequencies in using expressive suppression, psychological distress was significantly associated with more severe depression symptoms (β = 0.52, *p* < 0.001; Figure 2). Among individuals who reported a lower level of expressive suppression, significant correlation was also found between psychological distress and depression (β = 0.33, *p* < 0.001). Thus, psychological distress related to COVID-19 was associated with more severe symptoms of depression among participants both with high and low habitual usage of expressive suppression strategy, but with a greater predictive value among those who reported higher levels of suppression. Nevertheless, the interaction effect of cognitive reappraisal and psychological distress on depression was not significant (β = −0.02, *p* = 0.696); thus, the associations between psychological distress and depression severity were not influenced by cognitive reappraisal.

**Table 3:** Testing the moderated mediation effect of social media exposure, psychological distress, and expressive suppression on depression.

	**Psychological distress**			**Depression**		
	**β**	***SE***	***t***	**β**	***SE***	***t***
Social media exposure	0.24	0.04	5.43^**^	−0.04	0.04	−0.87
Psychological distress (PD)				0.43	0.04	10.12^**^
Expressive suppression (ES)				0.08	0.04	1.87
PD × ES				0.10	0.04	2.41^*^
*R*^2^	0.06			0.22		
*F*	29.47^**^			33.82^**^		

*N = 485. The beta values are standardized coefficients*.

* *p < 0.05*,

** *p < 0.01*.

## Discussion

In this study, we investigated the mediating role of psychological distress and the moderating role of emotion regulation on the relationship between social media exposure and symptoms of depression of the general public during the COVID-19 pandemic in China. The prevalence of depression was 15.9%, and depression severity was correlated with worse physical health. Older age and more frequent exposure to social media posts about COVID-19 were associated with a higher level of psychological distress. Moreover, psychological distress played a mediating role in the relationship between social media exposure and depression, and the associations between psychological distress and depressive symptom severity were moderated by expressive suppression. The results demonstrate the psychological impact of COVID-19 outbreak on non-patients and suggest targets for possible intervention programs for the general population.

In the current study, nearly one in six members of the general public reported at least mild depression. The prevalence rate in our sample was relatively lower than in previous studies, in which 20.1–53.5% of participants reported depressive and anxiety symptoms, respectively (10, 11), which was conducted from January 30 to February 13, during which the new confirmed cases of COVID-19 reached a peak, whereas the present study was conducted from February 16 to 19, during which time the number of recovered COVID-19 patients has exceeded that of new cases for the first time (55). Moreover, this discrepancy might be related to the different measures of depressive symptoms used in the three studies. The present study applied the BDI-II, which was constructed based on the cognitive–behavioral model and emphasizes the cognitive symptoms of depression (56). Huang and Zhao (10) applied the CES-D, which emphasizes negative emotions (12), and Liu et al. (11) applied the PHQ-9, which incorporates the Diagnostic and Statistical Manual of Mental Disorders, Fourth Edition (DSM-IV) diagnostic criteria for major depressive disorder (13). Lambert et al. (57) found that the PHQ-9 cutoff is easier to reach than the CES-D cutoff, and the CES-D cutoff score is easier to reach than the BDI-II cutoff. The present study was administered during the COVID-19 outbreak; it could be more convincing to measure the dependent variable by comparing the severity of depressive symptoms from before and during the pandemic. A nationwide epidemiological study, however, demonstrates a lifetime prevalence rate of 6.8% for depression disorders in China (58); thus, the prevalence of depressive symptoms is more than two-fold higher during the COVID-19 pandemic compared with before the COVID-19 pandemic.

In the present study, individuals with worse self-reported physical health also reported more elevated levels of depression and psychological distress about COVID-19. Although our participants were not infected by COVID-19, the rapid spread and high infectiousness of the virus (59) can cause changes in the lifestyles of non-patients, such as isolation to avoid exposure. Moreover, the practice of social distancing may result in more loneliness, which might contribute to elevated depressive symptoms (60). These lifestyle changes have been shown to have negative psychological effects, including generalized anxiety disorder, symptoms of depression, disrupted sleep (10), and symptoms of acute posttraumatic stress disorder (PTSD) (14).

People at an older age reported higher levels of psychological distress, which was consistent with Qiu et al. (9). The elderly and people with underlying health conditions have been shown to be more vulnerable to COVID-19 (61, 62). Perceived ageism and social isolation also contributed significantly to the relationship between age and psychological distress (63). Therefore, psychological interventions and physical health care services for the elderly are in urgent need to accommodate for potential emotional distresses in response to the COVID-19 crisis (64).

Informed by the emotional contagion theory and media effect theory, the study examined the association between social media exposure and psychological distress, and we found exposure to social media content concerning COVID-19 was associated with greater psychological distress. Indirect exposure to traumatic event *via* electronic media could lead to increased levels of PTSD and vicarious trauma (65, 66), especially exposure to the widely disseminated misleading information related to the COVID-19 outbreak on social media platforms (67). Additionally, the significant associations between social media exposure and depression severity were consistent with findings from a recent study, in which time spent on COVID-19 news *via* social media was utilized as measures of social media exposure, and they found that the time spent on social media was related to elevated depressive symptoms (68). Besides, the mediation effect suggested that social media exposure contributed to the elevated depressive symptom through psychological distress. Media exposure to COVID-19 has been found to be positively related to acute stress (69). There is considerable evidence that greater social media exposure is a risk factor contributing to depression and psychological distress in adolescents (70); further investigations are needed to clarify the potential moderators between the relationship of social media exposure and depressive severity related to COVID-19 in people of different ages.

Greater psychological distress related to COVID-19 was positively correlated with more severe depression symptoms. Psychological distress has been shown to be a common response to traumatic events such as traffic accidents and natural disasters (71, 72). Psychological distress has also been shown to be present nearly 4 years after receiving a diagnosis of SARS, an infectious disease that affects the respiratory system similar to the COVID-19 (73), suggesting a persistent impact of this kind of infectious disease on mental health. The results in the current study suggest that psychological distress related to the COVID-19 pandemic may predict the development of more severe chronic psychiatric illnesses, such as depression.

Results showed that the interaction between expressive suppression and psychological distress positively predicted depression severity, suggesting that habitual use of suppression strategy together with higher levels of psychological distress in response to COVID-19 outbreak contributes to the development of depression symptoms. The result was consistent with that of a recent study (41), which found that individuals who reported a higher level of expressive suppression exhibited enhanced physiological response in reaction to stressful life events. A large amount of research has shown that expressive suppression was closely related to the development and maintenance of depression episodes (32, 74–77). Specifically, the usage of expressive suppression was associated with increased negative affect and decreased positive affect in daily life (78) and to be inconducive to the maintenance of good interpersonal relationships, thus aggravated depressive symptoms (79).

On the other hand, the associations between depression and cognitive reappraisal, an adaptive emotion regulation strategy, did not reach significance level. The result was consistent with those of previous research (80, 81), in which insignificant correlations between cognitive reappraisal and depression were reported. Contrary to expressive suppression, a response-focused emotion regulation, cognitive reappraisal was an antecedent-focused strategy, which requires individuals to make adjustments before behavior and psychological well-being are affected (32). The COVID-19 was a public health emergency of international concern; thus, it was difficult for individuals to pre-evaluate the psychological impact and to regulate their emotions ahead of its sudden outbreak. In addition, it has been shown that expressive suppression was associated with higher stress-related symptoms in trauma-exposed community samples, while cognitive reappraisal was not (40). The meta-analysis indicated a medium effect size on the associations between suppression and posttraumatic stress symptoms, but no significant effect was found for reappraisal and post-trauma symptoms (82). These findings indicated that for stress-related symptoms, expressive suppression may play a more important role than cognitive reappraisal. However, further studies are needed to test the potential mediating role of other emotion regulation strategies (such as distraction and social sharing) as well as consider other relevant outcome variables, such as anxiety.

The current study has several limitations. Firstly, the sample size was not large enough to be representative of non-patients affected by COVID-19 in China. Secondly, due to lockdown measures, data were collected *via* SNSs with self-reported questionnaires; thus, the results might be susceptible to memory bias and response tendencies such as social desirability. Recruitment *via* SNSs might bias samples and result in underrepresentation of older individuals (83). There were only a few participants over the age of 60 in the present study; the geriatric age-group, however, has a higher risk of contracting the disease and greater prevalence of psychological distress related to COVID-19 (46). Thirdly, this was a cross-sectional survey research that only revealed correlational effect. Causal relationships among social media exposure and depression cannot be determined. Longitudinal research is warranted to explore the dynamic change in mental health during different stages of the COVID-19 pandemic and uncover the underlying mechanism on the development and maintenance of mental disorders.

## Conclusions

The present study contributes to the better understanding of the role of social media exposure to COVID-19 in amplifying psychological distress and mental health consequences. Older age, poor self-reported physical health, and higher exposure to social media content about the pandemic were risk factors for mental health problems. Psychological distress fully mediated the relationship between social media exposure and depression. Additionally, habitual use of expressive suppression interacting with levels of psychological distress about COVID-19 contributed to a higher level of depression. The results highlight the necessity of providing psychological assistance for the elderly, and individuals reported greater psychological distress and habitual use of suppression during the COVID-19 pandemic. The current study helps to inform evidence-based guidelines for minimizing psychological distress and promoting mental well-being during the global pandemic emergency.

## Data Availability Statement

The raw data supporting the conclusions of this article will be made available by the authors, without undue reservation.

## Ethics Statement

This study was reviewed and approved by Central China Normal University. All participants provided informed consent to having their anonymous data used for research. In addition, informed consent was obtained from teachers of middle school students before data collection.

## Author Contributions

Y-tZ and R-tL collected and analyzed the data and wrote the first draft of the paper. X-jS and MP commented significantly to the draft of the paper. XL generated the idea, designed and supervised the study, and wrote the first draft of the paper. All authors have contributed to and have approved the final text.

## Conflict of Interest

The authors declare that the research was conducted in the absence of any commercial or financial relationships that could be construed as a potential conflict of interest.
